# Diaphragmatic Ultrasonography as a Predictor of Extubation Success in Children: Systematic Review and Meta‐Analysis

**DOI:** 10.1002/ppul.71537

**Published:** 2026-02-23

**Authors:** Diego Santos de Oliveira, Guilherme Jorge Costa, Sheyla Suelle dos Santos Levy, Alexandre Magno Delgado

**Affiliations:** ^1^ Department of Physiotherapy Instituto de Medicina Integral Professor Fernando Figueira—IMIP Pernambuco Brazil; ^2^ Instituto de Medicina Integral Professor Fernando Figueira—IMIP Pernambuco Brazil

**Keywords:** children, diaphragm, extubation, ultrasonography, weaning

## Abstract

**Background:**

This study aimed to determine the diagnostic accuracy of the Diaphragmatic Thickening Fraction (DTF) and Diaphragmatic Excursion (DE) in predicting extubation success.

**Methods:**

This study was a systematic review with meta‐analysis of observational studies. We searched the MEDLINE/PubMed, Embase, LILACS, CINAHL, Cochrane Central, PEDro, Web of Science, and SCOPUS databases, with no restrictions on period or language. The risk of bias and quality of the studies were assessed using the Quality Assessment of Diagnostic Accuracy Studies (QUADAS‐2), Newcastle Ottawa Score, and GRADE tools. RevaMan version 5.4 was used for the meta‐analysis.

**Results:**

A total of 14 studies were included in the meta‐analysis, which included 657 patients. DTF showed low overall accuracy AUC 0.63 (0.57–0.69), but performed better in infants AUC 0.82 (0.74–0.89) with a mean difference of 11.92 (7.73–16.11) mm between success and failure. DE showed greater diagnostic accuracy, with AUC 0.72 (0.58–0.85) and a mean difference of 2.21 (1.44–2.98) mm in infants. Assessment by the left hemithorax is still limited. The extubation failure rate was 26.9%, and the success group had shorter mechanical ventilation (−4.5 days) and hospitalisation (−12.2 days) times.

**Conclusions:**

Diaphragm ultrasound shows promise in predicting extubation success in children, especially in the assessment of diaphragmatic excursion. The thickening fraction showed better accuracy in infants, and the right hemithorax was the most evaluated. However, the heterogeneity of the studies limits the interpretation of the findings.

## 1 Introduction

Invasive mechanical ventilation (IMV) can lead to ventilator‐induced diaphragmatic dysfunction (VIDD) [1]. VIDD is common in patients undergoing prolonged mechanical ventilation and can delay extubation, prolong hospitalisation and increase mortality [2, 3]. In young children, especially infants, the accessory respiratory muscles are not yet fully developed, which limits their ability to compensate for diaphragmatic dysfunction. In this age group, the diaphragm is primarily responsible for ventilation, making these patients more susceptible to muscle fatigue and extubation failure when its function is compromised [1, 2, 3].

Diaphragmatic ultrasound (DUS) is a promising, non‐invasive, and safe tool for assessing diaphragmatic function during ventilatory weaning [4, 5]. Structural and functional changes in the respiratory muscles can occur early after the onset of mechanical ventilation in children, reflecting the impact of critical illness on the respiratory muscle pump [6]. Reduced thickness of the diaphragm and expiratory muscles has been associated with an increased risk of extubation failure, highlighting the role of DUS in monitoring respiratory muscle function and early identification of dysfunction [4, 5, 6]. Ultrasound can aid in predicting extubation success by monitoring diaphragm morphology through parameters such as diaphragm thickness, diaphragmatic excursion (DE), and diaphragmatic thickening fraction (DTF) [4, 5, 6, 7].

Recently, a systematic review evaluated the accuracy of diaphragmatic ultrasound in predicting the success of extubation in preterm infants and infants. However, methodological limitations must be considered, such as the inclusion of heterogeneou nd small sample sizes, which may compromise the accuracy of estimates and the generalisation of findings to other age groups [8].

This review of prospective cohort studies aimed to evaluate the accuracy of diaphragmatic thickening fraction and diaphragmatic excursion in predicting extubation success in children.

## 2 Methods

This systematic review with meta‐analysis of observational studies followed the MOOSE guidelines [9]. This study is registered in PROSPERO (*International Prospective Register of Systematic Reviews*) under number CRD42024603027.

### 2.1 Literature Search Strategy

We searched the PubMed, EMBASE, SCOPUS, LILACS, CINAHL, COCHRANE CENTRAL, Web of Science, and PEDro databases until 17 March 2025. There were no restrictions on the publication period of the articles or the language. The search strategy combined Medical Subject Headings (MeSH) terms and free‐text terms. Details of the complete search strategy are described in Table S1.

### 2.2 Inclusion and Exclusion Criteria

We included prospective cohort studies of children aged between 1 month and 17 years who had undergone more than 24 h of IMV; children who underwent DUS before extubation. Studies of children with neuromuscular disease and encephalopathy were excluded, as were studies with inadequate data to create a 2 × 2 table.

### 2.3 Data Extraction

To identify any relevant research, two independent authors (O. D., M. A.) reviewed the titles and abstracts. The full texts of eligible articles that met the inclusion criteria were then acquired and evaluated independently by the two reviewers. Duplicate citations were removed. Any discrepancies were resolved through deliberation by a third reviewer (C. G.). All authors agreed on any discrepancies after discussion.

The information retrieved included methodological characteristics of the studies, such as patient age, number of participants, weaning criteria, measurements, and cut‐off values. The data were entered into Review Manager software for accuracy checking, and when necessary, the authors of the original studies were contacted for clarification of incomplete information.

### 2.4 Study Quality Assessment

The Newcastle‐Ottawa Scale (NOS) tool was used to assess the methodological quality of the included observational studies [10]. The NOS assigns scores in three domains: participant selection, comparability of groups, and assessment of outcomes [10]. The total score was used to classify methodological quality as high, moderate, or low [10]. The QUADAS‐2 tool was used to assess the risk of bias in diagnostic accuracy studies, covering the domains of patient selection, flow and time, index test, and gold standard [11]. The risk of bias was classified as low, high, or uncertain [12].

The quality of evidence was assessed using the GRADE system, considering factors such as methodological limitations, inconsistency of findings, direction of results, imprecision of estimates, and publication bias [13]. Evidence was classified into high, moderate, low, or very low categories [13].

### 2.5 Statistical Analysis

Heterogeneity between studies was assessed using the P test and the I² index, considered significant when *p* < 0.05 and classified as low if I² ≤ 30%. Due to the variability between studies, a random effects model was used. Studies without OR or 95% CI were considered suggestive of selective reporting. Results presented as median and interquartile range were converted to mean and standard deviation using the Cochrane calculator for standardisation and inclusion in the meta‐analysis.

The combined values of TP, FN, FP, and FN allowed the calculation of sensitivity, specificity, likelihood ratios (positive and negative), and diagnostic odds ratio, with 95% CI, in addition to ROC curve and AUC analysis. When absent, absolute data were estimated using formulas based on sensitivity, specificity, and number of patients/non‐patients [14]. Analyses were performed using Review Manager 5.4.

## 3 Results

### 3.1 Literature Search Results

The review followed the PRISMA flowchart for study selection, with a search conducted in eight databases, resulting in 907 records (Figure S1). After removing duplicates, 637 articles were identified. A total of 270 articles were screened, and after screening titles and abstracts, 256 were excluded, leaving 14 studies included after evaluation of the full texts [4, 5, 15, 16, 17, 18, 19, 20, 21, 22, 23, 24, 25, 26], totalling 657 patients, as detailed in Table 1.

**Table 1:** Characteristics of the studies included in the review.

Study	Country	*N*	Age (success or failure) in (%), mean (±SD), median (IQR)	Inclusion	Measures	Diaphragm	Extubation failure
Lee E‐P (2017) [4]	Taiwan	45	1,8 (0.5–5) vs 2 (1 −11)†	>24 h IMV	DTF, TIMV, LS	Right	Reintubation within 48 h after extubation
Dionisio MT (2019) [5]	Portugal	17	42 (9,5–155,5)*	>1 h IMV	DTF, DA	Right	Reintubation within 48 h after extubation
Xue Y (2019) [14]	China	50	36 (15–84) vs 42 (10–158)*	>48 h IMV	DTF, DE, DA, TIMV, LS	Right	Reintubation within 48 h after extubation
Mistri S (2020) [15]	Índia	35	7 (4–10) vs 9 (6,10)†	>24 h IMV	DTF, TIMV, LS	Right	Reintubation within 48 h after extubation
Abdel Rahman (2020) [16]	Egito	106	Infants 44 (52,4) vs 40 (47,6); Childrens 9 (90) vs 1 (10); Adolescents 11 (91,7) vs 1 (8,3)	Weaning from IMV	DTF, DE, DA, TIMV, LS	Right and left	Reintubation or need for NIV within 72 h after extubation
Montoro DV (2021) [17]	Espanha	45	3 (1–19)*	>48 h IMV	DTF, TIMV, LS	Right	Reintubation or need for NIV within 48 h after extubation
Subhash S (2021) [18]	Índia	26	32 (5,75–96)*	>24 h IMV	DTF, DA	Right	Reintubation or need for NIV within 48 h after extubation
Aslan G (2022) [19]	Turquia	40	Crianças 1 mês a 10 anos Children 1 month to 10 years	>48 h IMV	DTF, DE, DA, TIMV, LS	Right	Reintubation or need for NIV within 48 h after extubation
Yao Y (2022) [20]	China	72	23,7 (±6,2) vs 22,8 ± (6,8)*	>48 h IMV	DTF, DE, DA, TIMV, LS	Right and left	Failure of SBT or Reintubation or need for NIV within 48 h after extubation
Shah A (2023) [21]	USA	38	9,5 (4–36) vs 13,5 (5–22)*	>48 h IMV	DTF, DA, TIMV, LS	Right	Reintubation or need for NIV within 48 h after extubation
Vadivelu S (2023) [22]	Índia	40	5 (1–8,5) vs 0,33 (0,25‐1)†	>24 h IMV	DTF, DA, TIMV, LS	Right	Reintubation within 48 h after extubation
Duyndam A (2023) [23]	USA	53	3 (0,10–48) vs 2 (0,81–183)*	>48 h IMV	DTF, TIMV, LS	Right	Reintubation or need for NIV within 48 h after extubation. HFNC was not considered a failure.
Eskander EM (2024) [24]	Egito	30	45 (6,5–86‐75) vs 21,5 (8–55,25)*	>24 h IMV	DTF, DE, DA, TIMV, LS	Right and left	Failure of SBT or reintubation within 48 h after extubation.
Ge H (2024) [25]	China	45	3,64 (±2,28) vs 2,67 (±2,09)†	>48 h IMV	DTF, DE, DA, TIMV, LS	Right	Reintubation or need for NIV or HFNC within 48 h after extubation.

*Note:* *: age in months; †: age in years.

Abbreviations: DA, diagnostic accuracy; DE, diaphragmatic excursion; DTF, diaphragmatic thickening fraction; h: hours; HFNC, high‐flow nasal cannula; IMV, invasive mechanical ventilation; LS, length of stay in the intensive care unit; NIV, noninvasive ventilation; SBT, spontaneous breathing test; TIMV, time of invasive mechanical ventilation.

Of the 14 studies included, six evaluated DE [4, 5, 15, 17, 20, 21] and 14 evaluated DTF [4, 5, 15, 16, 17, 18, 19, 20, 21, 22, 23, 24, 25, 26], with three analysing both sides of the diaphragm [17, 21, 25]. Eight studies investigated the accuracy of DTF [5, 15, 17, 19, 20, 21, 22, 23] and five investigated the accuracy of DE in predicting extubation success [15, 17, 20, 21, 25]. Twelve reported the duration of IMV [4, 15, 16, 17, 18, 20, 21, 22, 23, 24, 25, 26] and nine reported the length of stay in the intensive care unit (ICU) [4, 15, 16, 17, 18, 20, 21, 22, 23, 24] (Table 1).

The criteria for weaning from invasive mechanical ventilation were defined according to the clinical and ventilatory parameters described in each included study [4, 5, 15, 16, 17, 18, 19, 20, 21, 22, 23, 24, 25, 26]. Successful extubation was defined mainly as the maintenance of spontaneous ventilation without the need for reintubation within 48 h after extubation [4, 5, 15, 16, 17, 18, 19, 20, 21, 22, 23, 24, 25, 26]. Only one study considered reintubation within 72 h as failure [17], while two defined failure as unsuccessful spontaneous breathing trial (SBT) [21, 25]. Eight studies also included the need for high‐flow nasal cannula [24] or non‐invasive ventilation (NIV) [17, 18, 19, 20, 21, 22, 24, 25, 26] as criteria for failure (Table 1).

### 3.2 Study Characteristics and Quality Assessment

**Figure 1:**
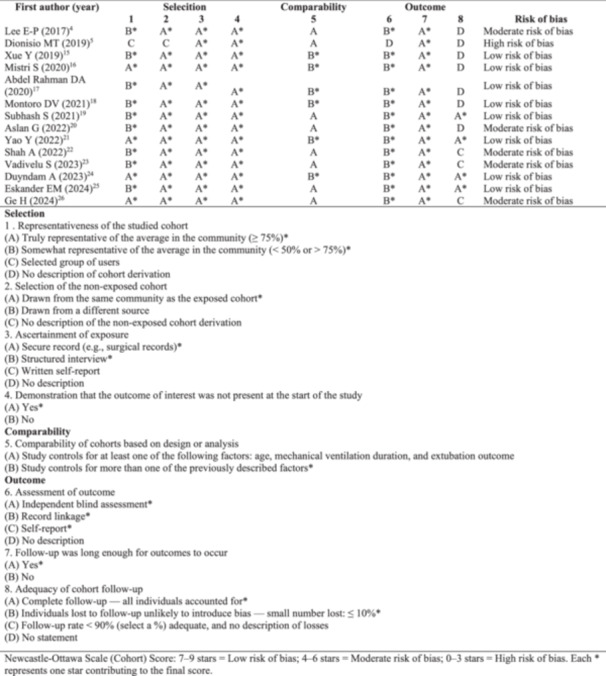
Risk of bias in cohort studies based on the Newcastle–Ottawa.

**Figure 2:**
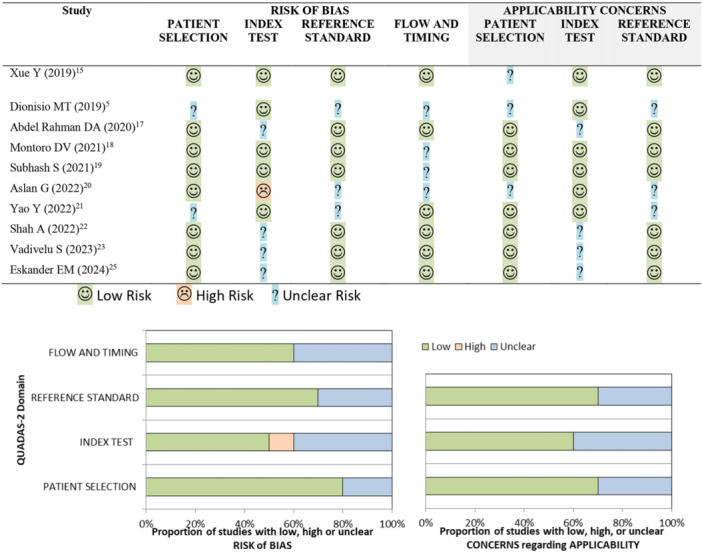
Risk of bias in assessment in diagnostic accuracy studies using the QUADAS Tool. [Color figure can be viewed at wileyonlinelibrary.com]

The quality of observational studies, assessed by the Newcastle‐Ottawa Scale (NOS) (Figure 1), raised concerns about selection bias and lack of adjustment to minimise confounding factors, such as varied extubation criteria and DUS measurements under different IMV settings, indicating caution in interpretation. The risk of bias in diagnostic accuracy studies, assessed by the QUADAS tool (Figure 2), showed low risk in patient selection (80%), low‐risk index test (50%), low‐risk reference standard (70%), and low risk in flow/timing (60%) of studies. The quality of evidence for the outcomes DTF, DE, mechanical ventilation time, and length of stay in the ICU was assessed by the GRADE system, considering methodological robustness and clinical applicability (Table 2) [12].

**Table 2:** Assessment of certainty of evidence using GRADE.

Oliveira DS, et al. Assessment of certainty of evidence using GRADE for outcomes of extubation success, duration of mechanical ventilation, and length of stay in the intensive care unit.						
Diaphragmatic ultrasonography as a predictor of extubation success in children						
Patient or population: Children undergoing invasive mechanical ventilation for more than 24 h						
Intervention: Diaphragmatic ultrasonography						
Comparison: Extubation success group versus extubation failure group						

Outcome N° of participants: (N° of NRS)	Relative effect (95% CI)	Anticipated absolute effects (95% CI)			Certainty	Importance
		Extubation failure	Extubation success	Diference		
DTF extubation success N° of participants: 594 (12 NRS)	RR 6.11 (2.20, 10.02)	100%	100%	511.0% more (120 more to 902 more)	⊕◯◯◯ Very low^a^^,^ b ^,^ c	Not important
DTF extubation success in infants N° of participants: 239 (3 NRS)	RR 11.92 (7.73, 16.11)	100%	100%	1092.0% more (673 more to 1.511 more)	⊕⊕⊕⊕ High^c^^,^ d ^,^ e	Important
DE extubation success N° of participants: 358 (6 NRS)	RR 0.86 (0.15, 1.57)	100%	100%	14.0% fewer (85 fewer to 57 more)	⊕◯◯◯ Very low^a^^,^ b ^,^ c	Not important
DE extubation success in infants N° of participants: 156 (2 NRS)	RR 2.21 (1.44, 2.98)	100%	100%	121.0% more (44 more to 198 more)	⊕⊕⊕⊕ High^c^^,^ d ^,^ e	Important
Left DTF extubation success N° of participants:208 (3 NRS)	RR 10.39 (5.93, 14.85)	100%	100%	939.0% more (493 more to 1.385 more)	⊕◯◯◯ Very low^c^^,^ f ^,^ g	Not important
Left DE extubation success N° of participants: 136 (2 NRS)	RR 1.33 (−0.10, 2.75)	100%	100%	33.0% more (110 fewer to 175 more)	⊕⊕◯◯ Low^c^^,^ f ^,^ g	Not important
Time of invasive mechanical ventilation N° of participants: 594 (12 NRS)	RR −4.50 (−6.64, −2.36)	100%	100%	550.0% fewer (764 fewer to 336 fewer)	⊕⊕◯◯ Low^a^^,^ b ^,^ c	Not important
Length of stay in intensive care unit N° of participants: 398 (9 NRS)	RR −12.22 (−18.88, −5.57)	100%	100%	1322.0% fewer (1.988 fewer to 657 fewer)	⊕⊕◯◯ Low^a^^,^ b ^,^ c	Not important

*Note:* *The risk in the intervention group (and its 95% CI) is based on the assumed risk in the comparison group and the relative effect of the intervention (and its 95% CI). CI, confidence interval; MD, mean difference; RR, risk ratio. GRADE Working Group grades of evidence. High certainty: we are very confident that the true effect lies close to that of the estimate of the effect. Moderate certainty: we are moderately confident in the effect estimate: the true effect is likely to be close to the estimate of the effect, but there is a possibility that it is substantially different. Low certainty: our confidence in the effect estimate is limited: the true effect may be substantially different from the estimate of the effect. Very low certainty: we have very little confidence in the effect estimate: the true effect is likely to be substantially different from the estimate of effect.

Abbreviations: DE, diaphragmatic excursion; DTF, Diaphragm fractional thickening; NRS, Non‐Randomised Studies.

Explanations

^a^ Methodological variations in weaning criteria and clinical differences, including the age range of participants.

^b^ There was righ inconsistency (I² ≥ 75%)

^c^ Wide confidence intervals.

^d^ Methodological variations in weaning criteria and clinical differences.

^e^ There was low inconsistency (I² < 40%, *p* > 0,05)

^f^ Methodological variations in weaning criteria and clinical differences, including the age range of participants. Also technical difficulties in obtaining images in the left hemithorax.

^g^ There was moderate inconsistency (I² ≥ 40%, *p* ≤ 0,05)

### 3.3 Technique and Window for Ultrasound Assessment of the Diaphragm, Weaning Criteria, and Extubation Failure

Diaphragmatic ultrasound was performed before or during SBT with different devices, including Logic E9 (GE), Honda HS‐2100, PHILIPS CX50 POC, Mindray M7, Sonosite M Turbo, Sonosite EDGE II, Esaote MyLab Omega, NextGen LOGIQ (GE Healthcare), Sonosite SII, Philips Lumify, and Samsung HM70A [4, 5, 14, 15, 16, 17, 18, 19, 20, 21, 22, 23, 24, 25]. Convex (4–9 MHz) and linear (3–16 MHz) probes were used, with the patient in a semi‐reclining position on the ICU bed [4, 5, 15, 16, 17, 18, 19, 20, 21, 22, 23, 24, 25, 26].

The linear probe was positioned in the 8th or 9th right intercostal space, perpendicular to the chest wall, visualising the diaphragm as a three‐layer structure [4, 5, 15, 16, 17, 18, 19, 20, 21, 22, 23, 24, 25, 26]. On the left side, although the ideal positioning uses the spleen as an acoustic window, difficulties in visualisation require adjustments that may compromise the accuracy of measurements [17, 21, 25]. The evaluation of the left diaphragm prioritised the exclusion of severe dysfunctions [17, 21, 25].

The studies used B and M modes [4, 5, 15, 16, 17, 18, 19, 20, 21, 22, 23, 24, 25, 26]. B mode allowed real‐time visualisation of the diaphragm thickness during the respiratory cycle, while M mode graphically represented its movement over time [14, 17, 20, 21, 25, 26]. The variation between the use of modes and the inclusion or exclusion of pleural membranes contributed to data inconsistency, with overestimation when included and underestimation when excluded [4, 5, 15, 16, 17, 18, 19, 20, 21, 22, 23, 24, 25, 26].

Diaphragm thickness was measured between the pleural and peritoneal lines, with some studies using the midpoint [4, 5, 15, 16, 17, 18, 19, 20, 21, 22, 23, 24, 25, 26]. This methodological variation has minimal impact on the thickening fraction [4, 5, 15, 16, 17, 18, 19, 20, 21, 22, 23, 24, 25, 26]. DTF was calculated as the percentage change in thickness between these moments, using the formula: DTF = [(Inspiratory thickness – Expiratory thickness)/Expiratory thickness] × 100 [4, 5, 15, 16, 17, 18, 19, 20, 21, 22, 23, 24, 25, 26]. For DE, a convex probe was used in the subcostal region and M mode to identify the points of greatest and least displacement of the diaphragm [15, 17, 20, 21, 25, 26]. Hemidiaphragm measurements were performed in three respiratory cycles [4, 5, 15, 16, 17, 18, 19, 20, 21, 22, 23, 24, 25, 26].

Three studies evaluated the diaphragm through the right and left windows [17, 21, 25], highlighting greater difficulty in visualising the left side due to intestinal gases and the acoustic window limited by the spleen [17, 21, 25]. Most studies focused on the right side, supported by evidence that there are no significant differences in measurements between the sides [4, 5, 15, 16, 17, 18, 19, 20, 21, 22, 23, 24, 25, 26]. Nevertheless, the minimum and maximum values of DTF and DE were analysed bilaterally, considering the possibility of unilateral dysfunction with contralateral compensation [17, 21, 25]. It is recommended to evaluate the left side to rule out severe dysfunction or absence of motility [17, 21, 25].

The studies presented variations in the criteria and cut‐off values for weaning [15, 16, 17, 18, 19, 20, 21, 22, 23, 24, 25, 26]. In general, the following were considered: recovery from the primary disease, adequate spontaneous breathing, effective coughing, adequate level of consciousness, haemodynamic stability with minimal vasoactive support, oxygenation index < 6, absence of excessive tracheal secretion, adequate gas exchange, PEEP < 8 cmH₂O, and FiO₂ < 60% [15, 16, 17, 18, 19, 20, 21, 22, 23, 24, 25, 26].

The SBT presented variations in protocols between studies [15, 16, 17, 18, 19, 20, 21, 22, 23, 24, 25, 26] (Table S4). The duration ranged from 30 to 120 min or was adjusted according to patient tolerance, reaching up to 12 h [15, 16, 17, 18, 19, 20, 21, 22, 23, 24, 25, 26]. The modalities included pressure support (PS), which was more frequent, and continuous positive airway pressure (CPAP) [15, 16, 17, 18, 19, 20, 21, 22, 23, 24, 25, 26]. SP ranged from 7 to 10 cm H₂O, adjusted to the diameter of the endotracheal tube, while CPAP ranged from 4 to 6 cm H₂O [15, 16, 17, 18, 19, 20, 21, 22, 23, 24, 25, 26]. Signs of intolerance included fatigue and respirator, discomfort, tachypnoea, desaturation, tachycardia, sweating, agitation, and blood gas changes (↑PaCO₂ or ↓PaO₂) [15, 16, 17, 18, 19, 20, 21, 22, 23, 24, 25, 26].

The definition of extubation failure varied between studies [4, 5, 15, 16, 17, 18, 19, 20, 21, 22, 23, 24, 25, 26]. Some considered weaning failure to be the interruption of SBT [17, 21, 25], others considered it to be the need for reintubation or the use of NIV within 48–72 h after extubation [17, 18, 19, 20, 21, 22, 23, 24, 25], while some did not classify the use of post‐extubation NIV as failure [4, 5, 15, 16].

### 3.4 Diaphragm Thickening Fraction as a Predictor of Tasuccessful Extubation in Children

**Figure 3:**
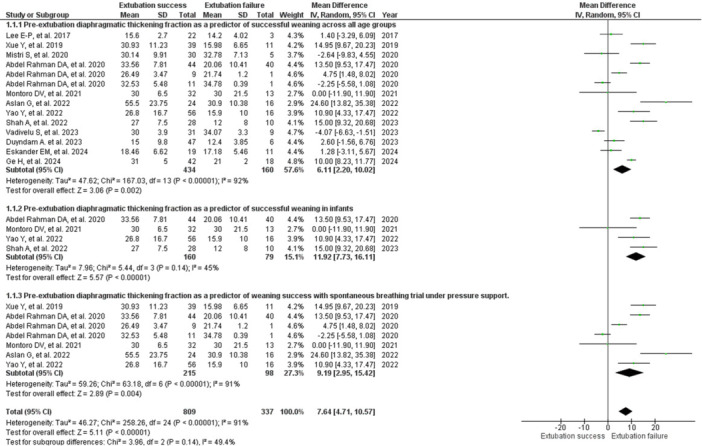
Forest Plot of diaphragm thickening fraction pre‐extubation as a predictor of successful weaning from invasive mechanical ventilation in children.

There was a 6.11% reduction in DTF in the group with extubation failure versus success (RR: 6.11; 95% CI: 2.20–10.2; 12 studies, 594 children) [4, 15, 16, 17, 18, 20, 21, 22, 23, 24, 25, 26] (Figure 3). The effect was significant for predicting success, but with very high heterogeneity (I²: 92%; T^(2)^: 47.62; *p* < 0.00001), possibly due to variations in weaning criteria and clinics between age groups [4, 15, 16, 17, 18, 19, 20, 21, 22, 23, 24, 25, 26]. The evidence was of very low certainty (Table 2).

The subgroup analysis of DTF in infants showed an 11.92% reduction in the group with extubation failure compared to success (RR: 11.92; 95% CI 7.73–16.11; four studies, 239 children) [17, 18, 21, 25]. There was a significant effect with moderate heterogeneity (I² 45%; T (^2)^ 7.96; *p* < 0.00001) and high certainty of evidence (Figure 3; Table 2). Meta‐analysis was not possible in preschoolers, schoolchildren, and adolescents because only one study was available [17].

There was a 6.11% reduction in DTF in the group with extubation failure versus success in the subgroup analysis that underwent SBT at support pressure (RR: 9.19; 95% CI: 2.95–15.42; five studies, 313 children) [15, 17, 18, 20, 21]. The effect was significant for predicting success, but with very high heterogeneity (I² 91%; T (^2)^ 59.26; *p* < 0.00001) (Figure 3).

### 3.5 Diaphragmatic Excursion as a Predictor of Successful Extubation in Children

**Figure 4:**
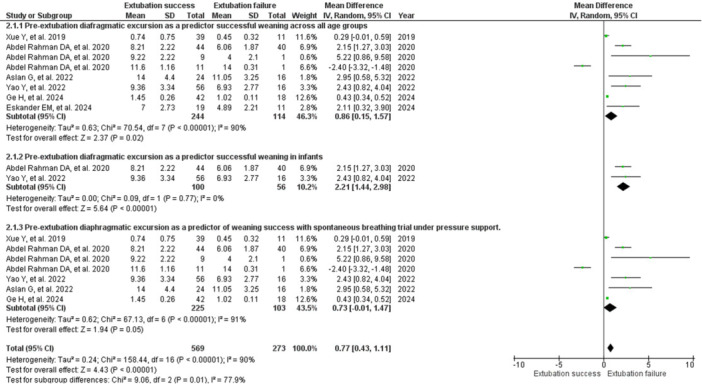
Forest Plot of pre‐extubation diaphragmatic excursion as a predictor of successful weaning from invasive mechanical ventilation in children. [Color figure can be viewed at wileyonlinelibrary.com]

There was a reduction of 0.86 mm in DE in the group with extubation failure compared to success (RR: 0.86; 95% CI: 0.15–1.57; 6 studies, 358 children) [15, 17, 20, 21, 25, 26]. There was a significant effect with very high heterogeneity (I²: 90%; T^(2)^: 0.63; *p* < 0.00001) and very low certainty of evidence (Figure 4; Table 2), possibly due to variations in extubation criteria and definition of failure.

In the subgroup analysis with infants, there was a 2.21 mm reduction in DE in the group with extubation failure compared to success (RR: 2.21; 95% CI: 1.44–2.98; 2 studies, 156 children) [17, 21]. The meta‐analysis was significant, with no heterogeneity (I²: 0%; T^(2)^: 0.00; *p* = 0.77), with high certainty of evidence (Figure 4; Table 2). Meta‐analysis was not possible in preschoolers, schoolchildren, and adolescents because only one study was available [17].

There was a 1.94 mm reduction in DE in the group with extubation failure versus success in the subgroup analysis that underwent SBT at support pressure (RR: 0.73; 95% CI: −0.01–1.47; five studies, 328 children) [15, 17, 20, 21, 25]. The effect was significant for predicting success, but with very high heterogeneity (I²: 91%; T^(2)^: 0.62; *p*: 0.05) (Figure 4).

### 3.6 Left Hemidiaphragm Thickening Fraction and Excursion as Predictors of Extubation Success in Children

There was a 10.39% reduction in the left diaphragm thickening fraction (LDTF) in the extubation failure group compared to the success group (RR: 10.39; 95% CI 5.93–14.85; 3 studies, 208 children) [17, 21, 25]. The meta‐analysis was significant, with high heterogeneit (I²: 72%; T^(2)^: 18.06; *p* = 0.006), with very low certainty of evidence (Figure S6; Table 2), possibly due to variations in weaning criteria and clinical differences, such as age group.

In the subgroup analysis with infants, there was a 15.08% reduction in LDTF in the group with extubation failure compared to success (RR: 15.08; 95% CI 3.23–26.94; two studies, 82 children) [17, 27]. The meta‐analysis was significant, with very high heterogeneity (I²: 86%; T^(2)^: 62.94; *p* = 0.008) (Figure S6), possibly related to technical difficulties in imaging the left hemithorax and variations in failure criteria [17, 25]. It was not possible to perform meta‐analyses for other age subgroups due to the limitation of studies [17].

There was a 1.33 mm reduction in left diaphragm excursion (LDE) in the group with extubation failure compared to success (RR: 1.33; 95% CI: −0.10–2.75; two studies, 136 children) [17, 25]. The meta‐analysis was significant, with very high heterogeneity (I²: 85%; T^(2)^: 1.67; *p* = 0.0001) and low certainty of evidence (Figure S7; Table 2), without sufficient evidence to conclude an effect of LDE as a predictor of success. Analysis by age subgroup was not possible because there was only one study [17].

### 3.7 Accuracy and Diagnostic Variability of Diaphragmatic Ultrasound in Predicting Extubation Success in Children

**Figure 5:**
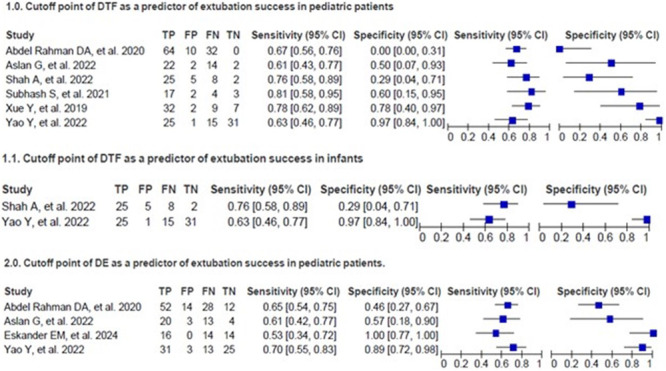
Florest plot cutt off DTF and DE pre‐extubation as a predictor of successful weaning from invasive mechanical ventilation in children. [Color figure can be viewed at wileyonlinelibrary.com]

Eight studies evaluated the DTF cutoff point in the outcome of extubation in children [5, 15, 17, 19, 20, 21, 22, 23]. Six identified values between 21% and 40% as predictors of success [15, 19, 20, 21, 22, 23], while two associated values between 23% and 35% with failure [5, 16] (Figure 5). Two studies focused on infants [21, 22], and two others did not present data on the diagnostic accuracy of DTF [5, 26].

Five studies presented DE cut‐off points for the outcome of extubation in children, ranging from 5.5 to 12.5 mm [15, 17, 20, 21, 25] (Figure 5). One study did not provide data on the diagnostic accuracy of DE [25].

**Figure 6:**
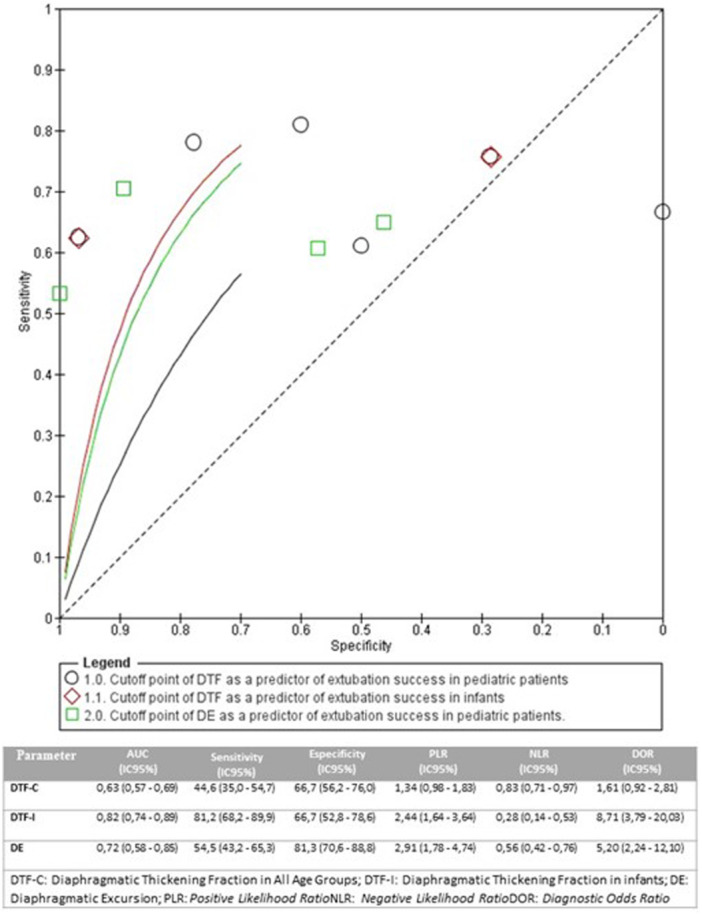
The area under the curve of summary receiver operating characteristic curves (AUSROC) DTF and DE. [Color figure can be viewed at wileyonlinelibrary.com]

The SROC analysis showed variability in the diagnostic performance of predictors of extubation success in children. DE in all age groups showed lower accuracy (AUC: 0.63; 95% CI: 0.57–0.69), with sensitivity of 44.6% and specificity of 66.7%, indicating limited performance in paediatric populations with heterogeneous age groups (Figure 6) [15, 17, 19, 20, 21, 22]. In contrast, DTF in the infant subgroup performed better (AUC: 0.82; 95% CI: 0.74–0.89), with a sensitivity of 81.2% and specificity of 66.7%, suggesting greater applicability in this age group (Figure 6) [21, 22].

DE as a predictor of successful extubation showed intermediate performance (AUC: 0.72; 95% CI: 0.58–0.85), with a sensitivity of 54.5% and specificity of 81.3%, indicating clinical utility, although with lower accuracy than DTF in infants (Figure 6) [15, 17, 20, 21, 25].

### 3.8 Mechanical Ventilation Time, Extubation Failure, Ventilator‐Associated Pneumonia, ICU Length of Stay, and Mortality in Children Evaluated by Diaphragmatic Ultrasound

There was a reduction of 4.5 days in IMV in the successful extubation group compared to the failure group (RR: −4.50; 95% CI: −6.64 to −2.36; 12 studies, 594 children) [4, 15, 16, 17, 18, 20, 21, 22, 23, 24, 25, 26]. The meta‐analysis was significant, with very high heterogeneity (I²: 81%; T^(2)^: 8.30; *p* < 0.0001) and low certainty of evidence (Figure S10; Table 2). The mean time was 9.29 ± 2 days in the successful group and 13.4 ± 8 days in the failure group. In the subgroup analysis with infants, there was a reduction of 1.37 days in invasive mechanical ventilation in the successful extubation group compared to the failure group (RR: −1.37; 95% CI: −3.06 to 0.33; three studies, 170 children) [18, 21, 22]. The meta‐analysis was not significant, with moderate heterogeneity (I²: 32%; T^(2)^: 0.78; *p* < 0.23) (Figure S10), suggesting a lack of consistent evidence of an association between diaphragmatic ultrasound assessment and reduced mechanical ventilation time.

There was a reduction of 12.22 days in ICU stay in the group with successful extubation compared to the group with failure (RR: −12.22; 95% CI −18.88 to −5.57; nine studies, 398 children) [4, 15, 16, 18, 20, 21, 22, 23, 24]. The meta‐analysis was significant, with very high heterogeneity (I²: 96%; T^(2)^: 80.61; *p* < 0.00001) and low certainty of evidence (Figure S11; Table 2), possibly related to variations in age range and clinical severity of patients. The meta‐analysis with the infant subgroup showed a reduction of 4.61 days of ICU stay in the successful extubation group compared to the failure group (RR: −4.61; 95% CI −10.98 to 1.75; two studies, 110 children) [21, 22]. The meta‐analysis was significant with very high heterogeneity (I^(2)^: 89%; T^(2)^: 18.87; *p*: 0.002) (Figure S11).

The extubation failure rate was 26.9% [4, 5, 15, 16, 17, 18, 19, 20, 21, 22, 23, 24, 25, 26]. Clear data on extubation failures related to upper airway obstruction have not been reported. No associated adverse events, such as ventilator‐associated pneumonia, were reported. Only three studies reported overall mortality (*n* = 92), with a mean of 12.3% [4, 5, 15].

## 4 Discussion

Diaphragm ultrasound has been widely used to predict extubation success in paediatrics [8]. In this meta‐analysis, the DTF showed a difference of 6.11 mm between the extubation success and failure groups, but with low diagnostic accuracy (AUC: 0.63). In infants, the mean difference was 11.92 mm, with better accuracy (AUC: 0.82), sensitivity of 81.2%, and specificity of 66.7% [8]. The low diagnostic accuracy of DTF may be related to methodological variability in studies regarding the inclusion of anatomical structures in the measurement, and factors such as the level of ventilatory support, PEEP, intrathoracic volume, and degree of sedation may influence DTF measurements.

Another important point to consider is the heterogeneity of the sample in terms of age, which included children aged 0 to 17 years, a period in which the diaphragm undergoes structural and functional changes involving the composition of muscle fibre, connective tissue, tendons, nerves, and vessels [27]. Diaphragm thickness and DTF tend to be greater in infants up to 1 year of age and decrease as the child's body surface area increases [28]. In addition, changes in the phenotype of myosin chains during growth increase muscle sSBTngth and endurance [98], which may influence respiratory capacity and susceptibility to respiratory failure, especially in young infants [20, 21, 22, 23, 24, 25, 26, 27, 28, 29, 30, 31].

Only six of the fourteen studies used SBT at support pressure, and the variation in PSV levels likely influenced inspiratory diaphragm thickening [15, 17, 18, 20, 21, 25]. Meta‐analyses showed reductions of 1.94 mm in DE and 6.11 mm in DTF among children who failed extubation, with significant effects to discriminate success. However, very high heterogeneity indicates significant methodological variability, especially related to the configuration of the support pressure level (dopted during the spontaneous breathing test. These findings suggest the clinical utility of diaphragmatic ultrasound, but reinforce the need for uniform protocols to increase the accuracy and comparability of results.

DE showed better accuracy than DTF in predicting extubation success. In the overall analysis, the mean difference was 0.86 mm (*p* = 0.02), and in infants, 2.21 mm (*p* < 0.00001), with AUC 0.72, sensitivity of 54.5% and specificity of 81.3% [15, 17, 20, 21, 25, 26]. This result may be related to the fact that DE reflects the functional capacity of the diaphragm and to the predominance of children over 2 years of age in the studies, in which excursion increases with age, while resistance to fatigue is greater at birth and decreases progressively [29, 30, 31, 32, 33]. In addition, diaphragmatic excursions may be influenced by factors such as the level of ventilatory support and PEEP, which modify the resting position and range of motion of the diaphragm [34, 35]. Although still controversial, DE has shown to be a promising predictor, especially in infants and preterm infants, with high sensitivity and specificity [17, 21, 32, 33].

The evaluation of the left diaphragm is often under‐explored due to technical difficulties in obtaining images, although there are no significant differences in the quality of visualisation compared to the right side, and it is useful for ruling out severe diaphragmatic dysfunction [17, 21, 25]. Despite the limitations of conventional ultrasound, the use of angle‐independent M‐mode can improve visualisation [17, 21, 25, 36, 37]. In addition, the evaluation of the highest point of the apposition zone with a high‐frequency linear transducer proved to be effective in both hemidiaphragms, highlighting the influence of the technique and equipment used on the accuracy of the ultrasound evaluation [21, 36].

Extubation failure in paediatric patients is a significant concern in ICUs, as it is associated with serious complications and higher mortality [38, 39, 40]. In this review, the failure rate was 26.9%, possibly influenced by the inclusion of patients under 24 months of age, with respiratory diseases or in the postoperative period of cardiac surgery. The main risk factors identified were age under 24 months, presence of respiratory and/or neurological diseases, respiratory muscle weakness, and cardiac condition, especially in the postoperative context [38, 39, 40, 41]. Upper airway obstruction is also recognised as the main cause of extubation failure in children [42]. However, in the included studies, the specific causes of failure were described heterogeneously, without a clear distinction between obstruction and muscle weakness, which should be considered when interpreting the results.

The consequences of extubation failure are associated with increased length of stay in the ICU, longer duration of mechanical ventilation, and greater need for tracheostomy, in addition to significantly increasing morbidity and mortality, especially in children with cardiac conditions [40, 41]. This review did not identify any studies that related extubation failure to invasive mechanical ventilation‐associated pneumonia in children evaluated by diaphragmatic ultrasound. A mean difference of −4.5 days in mechanical ventilation and −12.2 days in hospital stay was observed in favour of the group with successful extubation. The mortality rate, including patients with successful and failed extubation, was 12.3%.

The results of this meta‐analysis are imprecise due to the variability of IMV weaning protocols and the criteria used to define extubation failure, such as reintubation between 48 and 72 h or the need for non‐invasive ventilation after extubation. Currently, the decision to extubate children is based on daily assessment of standardised clinical and physiological criteria, aiming to avoid unnecessary prolonged ventilation [43, 44]. The assessment of readiness should consider cough sSBTngth, oropharyngeal secretion c, level of consciousness/sedation, and air leak test [7, 43, 44, 45, 46, 47].

The assessment of readiness for extubation should include SBT in CPAP, T‐piece, or PS modalities, with CPAP recommended for patients at higher risk of failure and PS for standard risk [43, 44, 45, 46, 47]. The ideal time for SBT is still debated, ranging from 30 min to 2 h, with evidence suggesting that 30 min may be sufficient [43, 44, 45, 46, 47]. During SBT, there is close monitoring to identify signs of intolerance [43, 44, 45, 46, 47]. Extubation failure in children is mainly defined by the need for reintubation within 48 h after extubation, although some definitions include the use of post‐extubation non‐invasive ventilation [40, 41, 42, 43, 44, 45, 46, 47].

Extubation failure in children is a multifactorial phenomenon related to challenges in oxygenation, ventilation, airway protection, and secretion management, as well as risk factors such as prolonged mechanical ventilation, prolonged ICU stay, sedation, neurological impairment, and underlying respiratory diseases [40, 41, 42, 43, 44, 45, 46, 47, 48, 49]. The decision to extubate should be based on a rigorous daily assessment that includes clinical and physiological criteria and specific tests, such as SBT [40, 41, 42, 43, 44, 45, 46, 47, 48, 49]. Therefore, this meta‐analysis suggests that DUS assessment, especially diaphragmatic excursion, emerges as a tool that can aid decision‐making, contributing to reduced failure rates and better clinical outcomes in paediatric patients.

The main limitations of the studies include the heterogeneity of clinical conditions and age groups, as well as variability in IMV weaning protocols, sedation levels, and ventilator settings during SBT. The level of ventilatory support, PEEP, intrathoracic volume, the level of sedation and respiratory muscle strength may influence DTF measurements and were not uniform between te studies. A análise da precisão diagnóstica foi limitada pela insuficiência de dados para construir tabelas 2 × 2, exigindo a estimativa de valores verdadeiros e falsos positivos e negativos, enquanto a heterogeneidade entre os estudos pode ter reduzido a consistência dos resultados.

Future studies should standardise protocols, criteria, and ultrasound techniques, in addition to performing stratified analyses by age group and in patients at high risk for extubation failure, with a clear description of accuracy data to improve the comparability of results.

## 5 Conclusion

Diaphragmatic ultrasound is a promising tool for predicting extubation success in children, with greater accuracy in DE assessment compared to DTF. Right‐sided assessment is the most common, with no significant differences compared to the left side, and is important for identifying severe diaphragmatic dysfunction.

## Author Contributions

**Diego Santos de Oliveira:** conceptualization, investigation, funding acquisition, writing – original draft, methodology, validation, visualization, writing – review and editing, formal analysis, data curation, software, project administration, resources. **Guilherme Jorge Costa:** conceptualization, investigation, writing – original draft, methodology, validation, visualization, writing – review and editing. **Sheyla Suelle dos Santos Levy:** conceptualization, investigation, writing – original draft, methodology, validation, visualization, writing – review and editing. **Alexandre Magno Delgado:** conceptualization, investigation, funding acquisition, writing – original draft, methodology, validation, visualization, writing – review and editing, formal analysis, data curation, software, project administration, resources.

## Ethics Statement

This is a systematic review with secondary data; approval by an ethics committee was not required.

## Conflicts of Interest

The authors declare no conflicts of interest.

## Supporting information


**Figure 01:** Flowchart of the study selection process. **Figure 06:** Forest Plot of left diaphragm thickening fraction pre‐extubation as a predictor of successful weaning from invasive mechanical ventilation in children. **Figure 07:** Forest Plot of left diaphragmatic excursion pre‐extubation as a predictor of successful weaning from invasive mechanical ventilation in children. **Figure 10:** Forest Plot of the time of invasive mechanical ventilation of children undergoing diaphragm assessment by ultrasound. **Figure 11:** Forest Plot of the length of stay in the intensive care unit of those undergoing diaphragm assessment by ultrasound. **Table 1:** Database search strategy. **Table 04:** Characteristics of spontaneous breathing tests and their interaction with diaphragmatic ultrasound parameters in the included studies.

## Data Availability

The data that support the findings of this study are available from the corresponding author upon reasonable request.
