# Supplementary material for: Diaphragmatic Ultrasonography as a Predictor of Extubation Success in Children: Systematic Review and Meta‐Analysis
Source: Pediatr Pulmonol. 2026 Feb 23;61(2):e71537. doi: 10.1002/ppul.71537 (PMC12927438; doi:10.1002/ppul.71537)
Supplement: Supplementary file 1 — Figure 01: Flowchart of the study selection process. Figure 06: Forest Plot of left diaphragm thickening fraction pre‐extubation as a predictor of successful weaning from invasive mechanical ventilation in children. Figure 07: Forest Plot of left diaphragmatic excursion pre‐extubation as a predictor of successful weaning from invasive mechanical ventilation in children. Figure 10: Forest Plot of the time of invasive mechanical ventilation of children undergoing diaphragm assessment by ultrasound. Figure 11: Forest Plot of the length of stay in the intensive care unit of those undergoing diaphragm assessment by ultrasound. Table 1: Database search strategy. Table 04: Characteristics of spontaneous breathing tests and their interaction with diaphragmatic ultrasound parameters in the included studies. [file PPUL-61-0-s001.docx]

**Table 1:** Database search strategy.

| **Database** | **Search strategy** |
| --- | --- |
| **MEDLINE (PUBMED)** | "ventilator weaning"[MeSH Terms] OR "weaning, ventilator"[All Fields] OR "mechanical ventilator weaning"[All Fields] AND "ultrasonography"[MeSH Terms] OR “ultrasonic Diagnosis”[Text Word] OR "diaphragm"[MeSH Terms] OR “respiratory diaphragm”[Text Word] AND “infant”[MeSH Terms] OR “children MeSH Terms] |
| **COCHRANE (CENTRAL)** | "ventilator weaning"[MeSH Terms] OR "weaning, ventilator"[All Fields] OR "mechanical ventilator weaning"[All Fields] AND "ultrasonography"[MeSH Terms] OR “ultrasonic Diagnosis”[Text Word] OR "diaphragm"[MeSH Terms] OR “respiratory diaphragm”[Text Word] AND “infant”[MeSH Terms] OR “children MeSH Terms] |
| **EMBASE** | "ventilator weaning" OR "weaning, ventilator" AND "ultrasonography" OR "diaphragm" AND “infant” OR “Children” |
| **LILACS** | (ventilator weaning) OR “respirator weaning”OR “mechanical, weaning” AND (diaphragm) OR "diaphragms, respiratory" AND (infant) OR (Children) |
| **CINAHL** | ("ventilator weaning") OR ("respirator weaning") OR“mechanical, weaning”AND“diaphragm” OR "diaphragms, respiratory" AND "infant” OR “Children” |
| **SCOPUS** | TITLE-ABS-KEY (ventilator weaning) OR TITLE-ABS-KEY (respirator weaning) AND TITLE-ABS-KEY (diaphragm) OR TITLE-ABS-KE (diaphragms, respiratory) ANDTITLE-ABS-KE (infant) OR (Children) |
| **Web of Science** | "ventilator weaning" OR "weaning, ventilator" AND "ultrasonography" AND "diaphragm" AND “infant” OR “Children” |
| **PEDro** | "ventilator weaning" OR "weaning, ventilator" AND "ultrasonography" AND "diaphragm" AND “infant” OR “Children” |

**Table 04.** Characteristics of spontaneous breathing tests and their interaction with diaphragmatic ultrasound parameters in the included studies.

| Study | Mode and duration of SBT | SBT configuration | DUS Assessment | PEEP/Volume/Insights | Measurement Techniques |
| --- | --- | --- | --- | --- | --- |
| Lee EP (2017) ^4^ | Clinical protocol (SIMV + PS); minimum of 12 hours. | FiO2 <30% and PEEP ≤6 mmHg. | Daily and immediately after extubation. | PEEP ≤6 mmHg. DTF <17% after extubation associated with failure. | B-mode for thickness measurement. Linear transducer 4-10 MHz. Average of three respiratory cycles. |
| Dionisio MT (2019) ^5^ | PSV ventilation mode; minimum of 23 hours. | PS in pre-extubation (mean maximum PIP of 16 ± 3.4). | Pre-extubation phase. | Maximum median PEEP of 6 cmH2O. TV maximum 7 ml/kg | B-mode for DTF and M-mode for DE. Linear transducer 10-5 MHz. |
| Xue Y (2019) ^15^ | SBT in PS; up to 30 minutes. | PS of 8 cmH2O and PEEP of 5 cmH2O | In the 5th minute after the start of SBT. | PEEP of 5 cmH2O. TV ≥5 mL/kg. DTF ≥21% predicts success with good sensitivity. | M-mode. Linear transducer (10 MHz) for thickness and convex transducer (5 MHz) for excursion. |
| Mistri S (2020) ^16^ | SBT in PS; duration not specified. | Minimum PS. | Daily, pre-extubation and within 12 hours post- extubation. | Median PEEP of 6. Post- extubation: diaphragm rises cranially, reducing thickness. | M-mode; 12-3 MHz transducer. Measurement of the muscle layer excluding pleural/peritoneal coverings. |
| Abdel Rahman (2020) ^17^ | SBT in PS; 30 to 120 minutes. | PS of 5 cmH2O | During SBT. | PEEP ≤5 cmH2O. Lung Score (pulmonary aeration) ≥12 predicts failure. | Linear transducer (9-11 MHz) for DTF and convex transducer (3.5 MHz) for M-mode excursion. |
| Montoro DV (2021) ^18^ | PSV ventilation mode; minimum of 12 hours. | Median PS 12 cmH2O (IQR 10-14). | pre-extubation phase or during PSV. | Median PEEP of 6 cmH2O during ventilation. Atrophy defined as a reduction >10% in thickness. | M-mode; 6-13 MHz linear transducer. Measured between the midpoint of the pleura and the peritoneum. |
| Subhash S (2021) ^19^ | SBT in PS; duration not specified. | PS of 5 cmH2O and PEEP of 5 cmH2O. | 30 minutes after the start of SBT, with the child calm. | PEEP of 5 cmH2O. TV of at least 5 mL/kg for SBT approval. | Linear transducer 6-13 MHz. Average of three consecutive measurements from the middle of the pleura to the middle of the peritoneum. |
| Aslan G (2022) ^20^ | ERT; up to 120 minutes. | Minimum pressure required to overcome the pressure in the tube; PEEP of 5 cmH2O. | At the end of the 2-hour ERT. | PEEP of 5 cmH2O. DTF and DE were the best predictors of failure. | Linear transducer (4-15 MHz) for thickness and convex transducer (6-10 MHz) for excursion. |
| Yao Y ( 2022) ^21^ | SBT in PS; up to 120 minutes. | PS of 5 cmH2O and PEEP of 5 cmH2O | Performed before the start of SBT (upon fulfilling weaning criteria). | PEEP of 5 cmH2O in the test. TV between 5-7 ml/kg. DE >8.08 mm predicts success. | Linear transducer (6-13 MHz) for thickness and convex transducer (2-5 MHz) for excursion. |
| Shah A (2022) ^22^ | SBT in PS; up to 120 minutes. | PS of 6 and PEEP of 5 cmH2O. | Within the first hour of SBT. | PEEP of 5 cmH2O fixed in the test. TV <5 mL /kg associated with SBT failure. | Linear transducer 4-12 MHz. B- mode for expiratory thickness; M-mode for DTF. |
| Vadivelu S (2023) ^23^ | SBT with unspecified mode and duration. | Maximum PEEP of 5 cmH2O. | Daily feeding for up to 14 days of mechanical ventilation and 3 days post- extubation. | PEEP of 5 cmH2O. High PEEP is correlated with a higher rate of atrophy. | M-mode; 6-13 MHz linear transducer. Measurement from the middle of the pleura to the center of the peritoneal line. |
| Duyndam A (2023) ^24^ | SBT in PS or CPAP; up to 120 minutes | PS of 10 cmH2O above PEEP 5; CPAP with PEEP 5. | Start SBT in support mode; optionally on CPAP before extubation. | PEEP of 5 cmH2O. TV between 5-7 ml/kg. No significant association between DTF and extubation success in this study. | Use of B-mode (2D). Measurement of muscle only within the fascial lines (current consensus). |
| Eskander EM (2024) ^25^ | SBT on PS; until 12 noon. | PS of 5 cmH2O. | During SBT. | PEEP ≤5 cmH2O. Long SBT (up to 12h) allows the diaphragm to recover function. | Microconvex transducer (4-9 MHz) used to visualize the left side (in 56.7% of cases). |
| Ge H (2024) ^26^ | SBT in PS; 120 minutes. | PS ≤7 cmH2O and PEEP ≤5 cmH2O | Before the start of SBT. | PEEP ≤5 cmH2O. Combined model (DTF, P/F ratio, PCIS) is superior to isolated indices. | Linear transducer (10 MHz). Excursion in B-mode; DTF in M-mode. |
| Differences in measurement techniques and PEEP:  • Zone of Apposition: Most studies use the zone of apposition, generally between the 8th and 10th intercostal spaces, because it more accurately reflects inspiratory force.  • Fascia vs. Muscle: Recent studies recommend measuring only the hypoechoic muscle portion, excluding the pleural and peritoneal fascia, in contrast to older studies that included the entire thickness between these structures.  • Impact of PEEP: PEEP maintains intrathoracic volume and functional residual capacity; its withdrawal during extubation can cause cranial displacement of the diaphragm, with elongation and thinning of muscle fibers on ultrasound.  • Positioning: The standardized technique recommends evaluation in the supine position, with the head of the bed elevated between 30 and 45 degrees. | | | | | |
| PSV: pressure support ventilation; PS: pressure support; DUS: diaphragmatic ultrasound; PEEP: positive end-expiratory pressure; cmH2O: centimeters of water; SBT: spontaneous bacterial tumor. breathing trial ; TV: tidal volume; mL /kg: milliliters per kilogram; DTF: diaphragmatic thickening fraction ; CPAP: continuous positive airway pressure; SIMV: synchronized intermittent mandatory ventilation; FiO2: fraction of inspired oxygen; mmHg: millimeters of mercury; DE: diaphragmatic excursion; PIP: peak inspiratory pressure; VM: mechanical ventilation; ERT: extubation readiness test ; Lung Score: pulmonary aeration score; MHz: megahertz ; 2D: two-dimensional; P/F ratio : PaO2/FiO2 ratio; PCIS: pediatric critical illness score. | | | | | |

**Figure 01.** Flowchart of the study selection process.


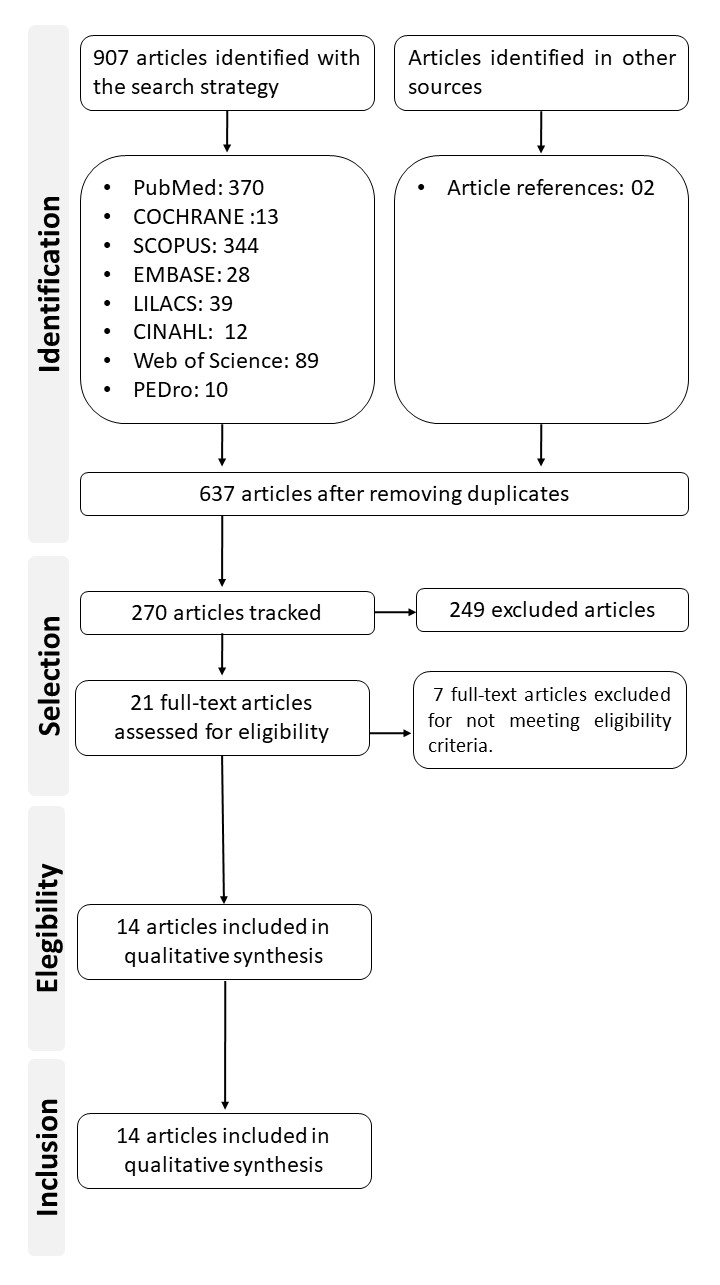


**Figure 06.** Forest Plot of left diaphragm thickening fraction pre-extubation as a predictor of successful weaning from invasive mechanical ventilation in children.


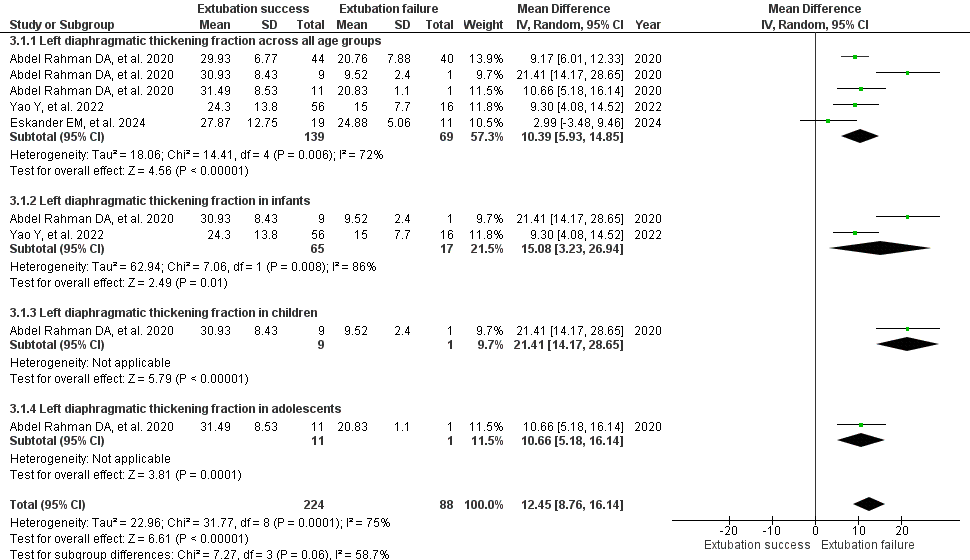


**Figure 07.** Forest Plot of left diaphragmatic excursion pre-extubation as a predictor of successful weaning from invasive mechanical ventilation in children.


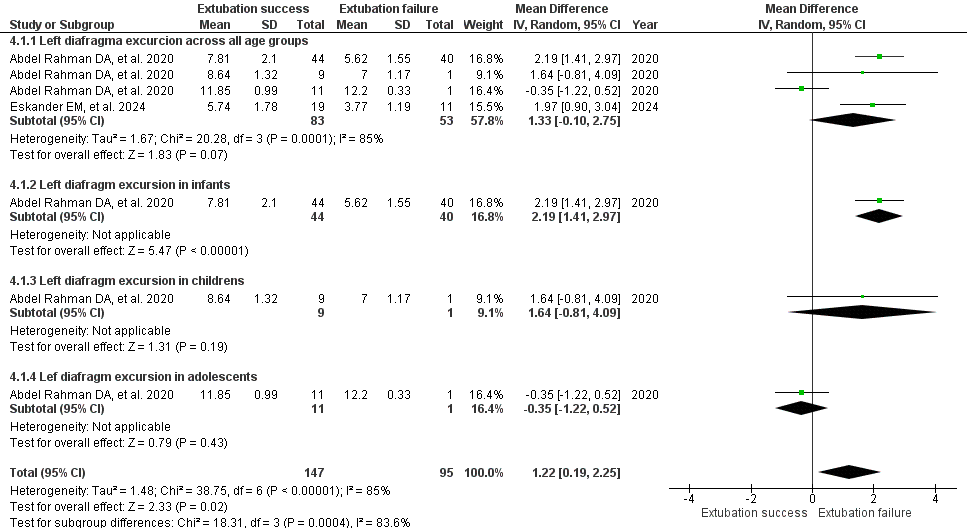


**Figure 10.** Forest Plot of the time of invasive mechanical ventilation of children undergoing diaphragm assessment by ultrasound.


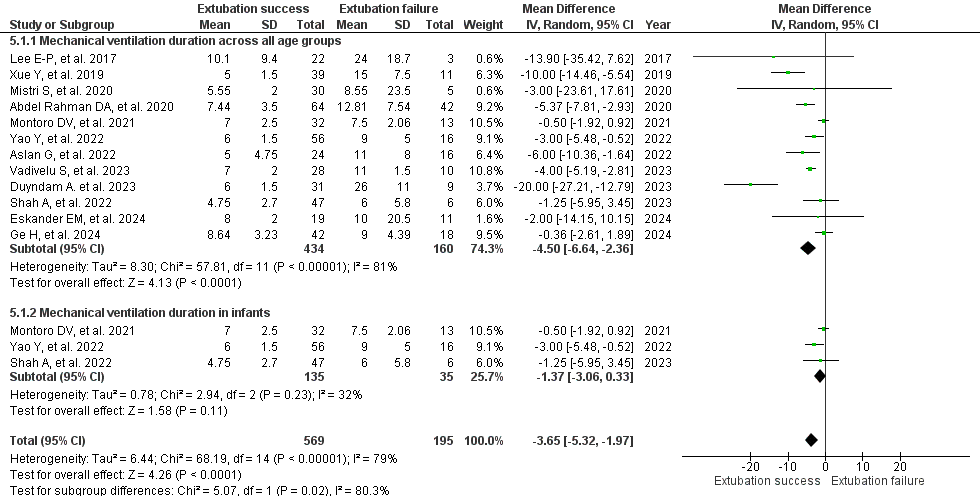


**Figure 11.** Forest Plot of the length of stay in the intensive care unit of those undergoing diaphragm assessment by ultrasound.

**
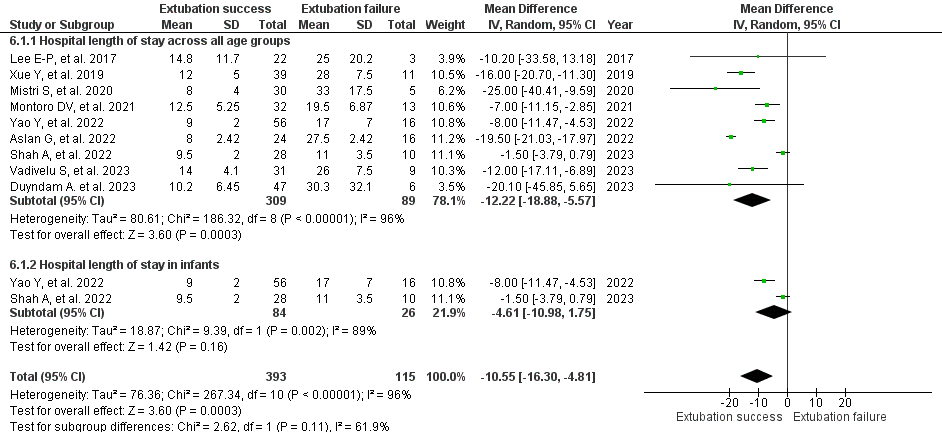
**

**List of Abbreviations**

| **Abbreviation** | **English Meaning** |
| --- | --- |
| AUC | Area Under the Curve |
| B-mode | Brightness Mode (ultrasound) |
| CAPES | Coordenação de Aperfeiçoamento de Pessoal de Nível Superior |
| CI | Confidence Interval |
| CPAP | Continuous Positive Airway Pressure |
| DE | Diaphragmatic Excursion |
| DOR | Diagnostic Odds Ratio |
| DTF | Diaphragm Thickening Fraction |
| DUS | Diaphragmatic ultrasound |
| ERT | Extubation readiness test |
| FiO₂ | Fraction of Inspired Oxygen |
| FN | False Negative |
| FP | False Positive |
| GRADE | Grading of Recommendations Assessment, Development and Evaluation |
| ICU | Intensive Care Unit |
| IMV | Invasive Mechanical Ventilation |
| LDE | Left Diaphragmatic Excursion |
| LDTF | Left Diaphragm Thickening Fraction |
| MeSH | Medical Subject Headings |
| M-mode | Motion mode (ultrasound) |
| NIV | Non-Invasive Ventilation |
| NLR | Negative Likelihood Ratio |
| NOS | Newcastle-Ottawa Scale |
| OR | Odds Ratio |
| PaCO₂ | Partial Pressure of Carbon Dioxide |
| PaO₂ | Partial Pressure of Oxygen |
| PEEP | Positive End-Expiratory Pressure |
| P/F | PaO2/FiO2 ratio |
| PLR | Positive Likelihood Ratio |
| PS | Pressure Support |
| PSV | Pressure support ventilation |
| PUCI | Pediatric Intensive Care Unit |
| QUADAS-2 | Quality Assessment of Diagnostic Accuracy Studies, version 2 |
| ROC | Receiver Operating Characteristic |
| RR | Risk Ratio |
| SBT | Spontaneous Breathing Trial |
| SCOPUS | Elsevier Abstract and Citation Database |
| SMIV | Synchronized intermittent mandatory ventilation |
| TN | True Negative |
| TP | True Positive |
| TV | Tidal volume |
| VAP | Ventilator-Associated Pneumonia |
| VIDD | Ventilator-induced diaphragmatic dysfunction |
| VM | Mechanical ventilation |
